# Transcriptome profiling of the initial segment and proximal caput of mouse epididymis

**DOI:** 10.3389/fendo.2023.1190890

**Published:** 2023-05-31

**Authors:** Xiao Wang, Fanyi Qiu, Junjie Yu, Meiyang Zhou, Anjian Zuo, Xiaojiang Xu, Xiao-Yang Sun, Zhengpin Wang

**Affiliations:** ^1^ Shandong Provincial Key Laboratory of Animal Cell and Developmental Biology, School of Life Sciences, Shandong University, Qingdao, China; ^2^ Department of Bioinformatics, Wanhui Biomedicine Co., LTD., Hangzhou, China; ^3^ Department of Pathology and Laboratory Medicine, Tulane University School of Medicine, Tulane University, New Orleans, LA, United States

**Keywords:** mouse epididymis, initial segment, proximal caput, sperm maturation, RNA-seq analysis

## Abstract

**Background:**

The proximal region of the mouse epididymis plays a pivotal role in sperm transport, sperm maturation, and male fertility. Several studies have focused on segment-dependent gene expression of the mouse epididymis through high-throughput sequencing without the precision of the microdissection.

**Methods and results:**

Herein, we isolated the initial segment (IS) and proximal caput (P-caput) by physical microdissection using an *Lcn9*-*cre*; *Rosa26^tdTomato^* mouse model. We defined the transcriptome changes of caput epididymis by RNA sequencing (RNA-seq), which identified 1,961 genes that were abundantly expressed in the IS and 1,739 genes that were prominently expressed in the P-caput. In addition, we found that many differentially expressed genes (DEGs) were predominantly or uniquely expressed in the epididymis and region-specific genes were highly associated with transport, secretion, sperm motility, fertilization, and male fertility.

**Conclusion:**

Thus, this study provides an RNA-seq resource to identify region-specific genes in the caput epididymis. The epididymal-selective/specific genes are potential targets for male contraception and may provide new insights into understanding segment-specific epididymal microenvironment-mediated sperm transport, maturation, and male fertility.

## 1 Introduction

In rodents, spermatozoa become mature and acquire their capacity to fertilize ova during epididymal transit. The epididymis is a segmented organ divided into three unique anatomical regions: caput, corpus, and cauda (1, 2). Each region plays a distinct role in concentrating sperm as well as sperm maturation and storage (3–5). Previous investigations have used the three regions as boundaries for analyses of gene and protein expression patterns (6, 7). In determining region-specific gene and protein expression, the epididymal regions have been further subdivided into intraregional segments separated by connective tissue septae (1, 8–11). Each of these segments possesses a distinct transcriptome that contributes to the different physiological functions of the segments during sperm maturation in the epididymis (12–14). The obvious functions of the epididymis from the initial segment (IS) to the cauda epididymis mainly include sperm transport, concentration, protection, motility acquisition, fertilizing ability acquisition, and storage. The IS is responsible for absorbing ~90% of fluid by epithelial cells to concentrate sperm from the rete testis. Additional absorption happens throughout the remainder of epididymal transition. After a series of maturation processes, 50%–80% sperm in the epididymal lumen are located and stored in the cauda epididymis prior to ejaculation. Thus, the epididymis is an essential reproductive organ responsible for sperm maturation in mammals.

The epididymal epithelium that lines the lumen is composed of multiple cell types including principal cells, basal cells, apical cells, clear cells, narrow cells, and halo cells (15–17). Distinct cell types within different epididymal regions are responsible for the region-specific luminal microenvironment that promotes early and late maturation of spermatozoa and subsequent storage in the cauda epididymis (18, 19). Impaired epididymal spermatozoa maturation causes dysfunction and failed fertilization (20). Thus, investigations of spermatozoa maturation in the epididymis provide insight into molecular mechanisms that support male fertility in mammals.

The caput epididymis is composed of an IS, the proximal caput (P-caput), and the distal caput. Studies have documented that different cell types and distinct regions of the caput epididymis express different genes that contribute to physiological functions during sperm transit (12, 21, 22). The IS has a wide luminal diameter and a low concentration of spermatozoa and is lined with elongated epithelial cells endowed with an abundance of stereocilia. The lumen rapidly narrows in the remainder of the caput, which increases the concentration of spermatozoa.

A series of studies have focused on the transcriptome analysis in the epididymis. However, they have been limited in conventional segmentation approaches. Here, the *Lcn9*-*cre*; *Rosa26^tdTomato^* mouse model facilitated precise microdissection of the IS and P-caput. This experimental approach allowed us to obtain more accurate segments and their specific transcriptomes. The current work aims to elucidate and compare transcriptomes in the IS and the P-caput of the epididymis. Using *Lcn9*-*cre*; *Rosa26^tdTomato^* mice to mark the boundaries between IS and P-caput, the IS and P-caput were precisely isolated by physical microdissection and their transcriptomes defined by RNA sequencing. The analyses established distinct transcriptional profiles for the IS/P-caput and provide powerful resources for future studies defining the functions of epididymis-selective/specific genes in spermatozoa maturation and male fertility.

## 2 Materials and methods

### 2.1 Animals

All animal procedures were approved by the Ethics Committee for Animal Research of School of Life Sciences, Shandong University, China. All experiments with mice were performed in accordance with guidelines of the care and use of laboratory animals. The *Lcn9-cre* mouse line and *Rosa26^tdTomato^* mouse line were kindly provided by Prof. Xiao-Yang Sun (23). *Rosa26^tdTomato^* females were crossed with *Lcn9-cre* males to generate *Lcn9-cre*; *Rosa26^tdTomato^* males.

### 2.2 Mouse genotyping

Mouse tails were lysed in DirectPCR Lysis buffer with proteinase K at 55°C overnight. To inactivate proteinase K, the samples were incubated at 85°C for 1 h. EmeraldAmp GT PCR Master Mix and primers were used to amplify specific DNA fragments. PCR was performed with an annealing temperature of 58°C and 35 cycles using Mastercycler Pro (Eppendorf). Primers for genotyping are listed in **Supplementary Table S1**.

### 2.3 Microdissection of IS and P-caput of mouse epididymis

Epididymides were collected from 3-month-old *Lcn9-cre*; *Rosa26^tdTomato^* males. The tdTomato signals were examined with a fluorescence stereomicroscope. The IS region displayed strong red fluorescence and could be accurately isolated by a 26G needle mounted on a 1-ml syringe. The P-caput adjacent to the IS was defined by connective tissue septae and isolated using the same size needle of 1-ml syringe. Two IS or P-caput samples from one male mouse were pooled as one replicate. RNA-seq analysis was performed on three biological replicates of IS or P-caput samples.

### 2.4 Immunofluorescence analysis

Epididymides were collected from *Lcn9-cre*; *Rosa26^tdTomato^* males, and tdTomato signals were examined with a fluorescence stereomicroscope. For immunostaining, freshly isolated epididymides from *Lcn9-cre*; *Rosa26^tdTomato^* mice were fixed in 4% PFA and embedded in Tissue-Tek OCT. Frozen sections were washed with PBS, incubated with Hoechst 33342 (Solarbio, 10 ug/ml) at RT for 5 min and mounted on the slides. Images were captured by a fluorescence microscope (Nexcope NE950).

### 2.5 RNA isolation and quantitative real-time RT-PCR

Total RNA was isolated from IS and P-caput of 3-month-old mouse epididymides using AFTSpin Tissue/Cell Fast RNA Extraction Kit for Animal (ABclonal) and cDNA was synthesized with SuperScript IV First-Strand Synthesis System (Thermo Fisher Scientific). Quantitative RT-PCR was performed using iTaq Universal SYBR Green Supermix (Bio-Rad) and the QuantStudio 6 Flex Real-Time PCR System (Thermo Fisher Scientific). The primers used in these experiments are listed in **Supplementary Table S1**. The relative abundance of each transcript was calculated by the 2^−ΔΔCt^ normalized to endogenous *β-actin* expression (24).

### 2.6 RNA-seq library preparation

Briefly, mRNA was purified from total RNA using poly-T oligo-attached magnetic beads. Fragmentation was carried out using divalent cations under elevated temperature in NEBNext First Strand Synthesis Reaction Buffer. First-strand cDNA was synthesized using random hexamer primer and M-MuLV Reverse Transcriptase. Second-strand cDNA synthesis was subsequently performed using DNA Polymerase I and RNase H. Remaining overhangs were converted into blunt ends *via* exonuclease/polymerase enzymes. After adenylation of 3’ ends of DNA fragments, the NEBNext Adaptor with a hairpin loop structure was ligated to prepare for hybridization (New England Biolabs). To select cDNA fragments of 250–300 bp, the library was purified with AMPure XP beads (Beckman Coulter). Then, 3 µl of USER Enzyme (New England Biolabs) was used with size-selected, adaptor-ligated cDNA at 37°C for 15 min followed by 5 min at 95°C before PCR. PCR was performed with Phusion High-Fidelity DNA polymerase, Universal PCR primers and Index Primer. PCR products were purified, and library quality was assessed on the Agilent 2100 system.

### 2.7 Sample clustering and sequencing

The clustering of the index-coded samples was performed on a cBot Cluster Generation System using TruSeq PE Cluster Kit v3-cBot-HS (Illumina) according to the manufacturer’s instructions. After cluster generation, the library preparations were sequenced on an Illumina Hiseq platform and 125-bp/150-bp paired-end reads were generated.

### 2.8 RNA-seq analysis

Reference genome and gene model annotation files were downloaded from the genome website directly. Clean paired-end reads were aligned to the reference genome using STAR. HTseq was used to count the reads numbers mapped to each gene. FPKM of each gene was calculated based on the length of the gene and read count mapped to this gene. Differential expression analysis was performed using the DESeq2 R package. Genes with an adjusted *p*-value <0.05 and absolute log2 (fold change) >1 by DESeq2 were assigned as differentially expressed. Published RNA-seq datasets (GSE181426) were analyzed using the same criteria for comparisons.

### 2.9 GO, KEGG, and PPI analysis

Gene Ontology (GO) and Kyoto Encyclopedia of Genes and Genomes (KEGG) enrichment analysis of differentially expressed genes (DEGs) was performed by the clusterProfiler R package. Protein–protein interaction (PPI) analysis of DEGs was based on the STRING database.

### 2.10 Statistical analysis

Data are presented as the mean ± SD. Statistical analysis was performed using GraphPad Prism. The variances of the two groups were compared by the two-tailed Student’s *t*-test, and significance was defined as *p < 0.05, **p < 0.01, ***p < 0.001, and ****p < 0.0001.

## 3 Results

### 3.1 Microdissection of initial segment and proximal caput

**Figure 1:**
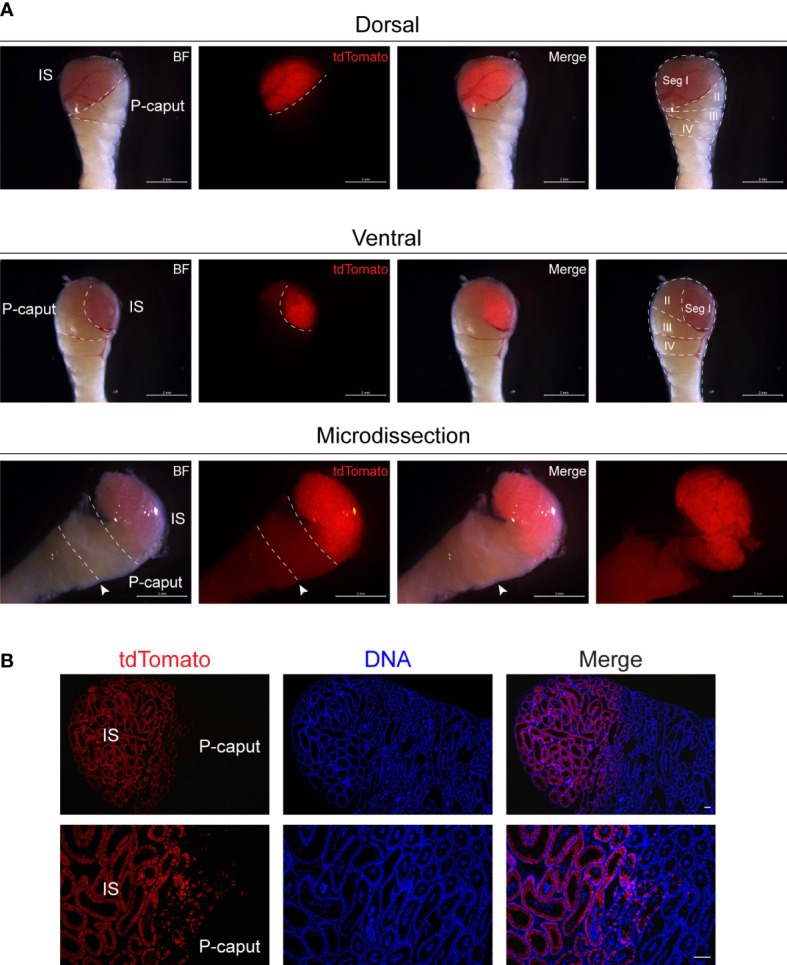
Microdissection of the IS and P-caput. **(A)** IS and P-caput were isolated from adult *Lcn9-cre*; *Rosa26^tdTomato^* mice under a fluorescence stereomicroscope. White dashed lines distinguished the boundaries between the IS and P-caput. Arrowheads indicated the connective tissue septae. Scale bar, 2 mm. **(B)** The proximal region-specific distribution of tdTomato signals detected in proximal epididymis with Cre recombination. Scale bar, 50 μm. IS, initial segment; P-caput, proximal caput; BF, brightfield.

To isolate the IS and P-caput in the adult mouse epididymis, we used *Lcn9-cre*; *Rosa26^tdTomato^* mice. *Lcn9-cre*; *Rosa26^tdTomato^* males were generated by crossing *Rosa26^tdTomato^* females with *Lcn9-cre* males in which the recombinase is specifically expressed in the IS as early as post-natal day 17 (P17) (23). Adult epididymides from *Lcn9-cre*; *Rosa26^tdTomato^* mice were dissected under a fluorescence stereomicroscope in combination with the histology-based epididymal segment determination method (12). As shown in **Figure 1A**, the IS region displayed strong red fluorescence and could be accurately isolated by a needle mounted on a 1-ml syringe. The P-caput adjacent to the IS was defined by connective tissue septae. To substantiate the expression pattern of tdTomato signals in the epididymis, immunofluorescent staining was analyzed using frozen sections from adult *Lcn9-cre*; *Rosa26^tdTomato^* males. The results confirmed specific staining of tdTomato in the IS (**Figure 1B**), consistent with previous findings (23). These data suggest that we successfully and accurately isolated the IS and P-caput in mouse epididymides.

### 3.2 Transcriptome analysis of IS and P-caput

**Figure 2:**
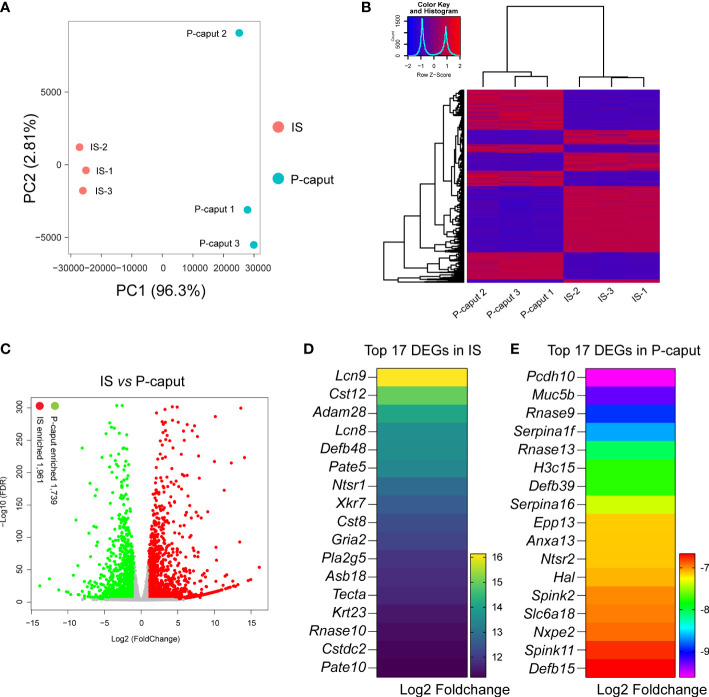
RNA-seq analysis of the IS and P-caput. **(A)** Principal component analysis (PCA) of RNA-seq data for the IS and P-caput (*n* = 3 independent IS samples, *n* = 3 independent P-caput samples). **(B)** Heatmap of differentially expressed genes (DEGs) between the IS and P-caput. **(C)** Volcano plot showing differential gene expression using an adjusted *p*-value <0.05 and log2 (fold change) >1 by DESeq2. **(D, E)** Expression patterns of the top 17 DEGs in IS **(D)** and the top 17 DEGs in P-caput **(E)** according to RNA-seq data.

To define region-specific transcriptomes of the proximal region of the epididymis, we performed bulk RNA-seq and compared the results between IS and P-caput. First, sample clustering by principal component analysis (PCA) and heatmap of DEGs showed that the three biological replicates in each group were very similar and the differences between the two groups were significant (**Figures 2A, B**), indicating that the data were reliable. Heatmap and volcano plots demonstrated distinct transcriptional patterns between IS and P-caput and identified a series of region-specific genes (**Figures 2B, C**). RNA-seq results identified 3,700 DEGs with 1,961 transcripts significantly enriched in the IS and 1,739 transcripts enriched in the P-caput using an adjusted *p*-value <0.05 and log2 (fold change) >1 by DESeq2 (**Figure 2C**). Based on the data, we identified a series of region-specific genes. The top 17 DEGs specifically expressed in the IS (**Figure 2D**) included *Lcn9*, *Cst12*, *Adam28*, *Lcn8*, *Defb48*, *Pate5*, and *Ntsr1*. While some DEGs (e.g., *Pcdh10*, *Muc5b*, *Rnase9*, *Serpina1f*, *Rnase13*, *H3c15*, and *Defb39*) were also primarily expressed in the P-caput, the top 17 DEGs in P-caput are listed in **Figure 2E**. We next performed Kyoto Encyclopedia of Genes and Genomes (KEGG) analysis to identify the enriched signaling pathways in the IS and P-caput regions. Glycosphingolipid biosynthesis, fatty acid biosynthesis, cAMP signaling pathway, calcium signaling pathway, pyrimidine metabolism, and glycerolipid metabolism were enriched in the IS (**Supplementary Figure S1A**). Phenylpropanoid biosynthesis, arginine biosynthesis, glycolysis, pyruvate metabolism, glycine, serine, and threonine metabolism were enriched in the P-caput (**Supplementary Figure S1B**). The STRING database was used to construct PPI networks of all and of the top 20 DEGs in IS, as well as all and the top 20 DEGs in P-caput (**Supplementary Figures S2A–D**). The PPI networks indicated potential interactions among the DEGs.

**Figure 3:**
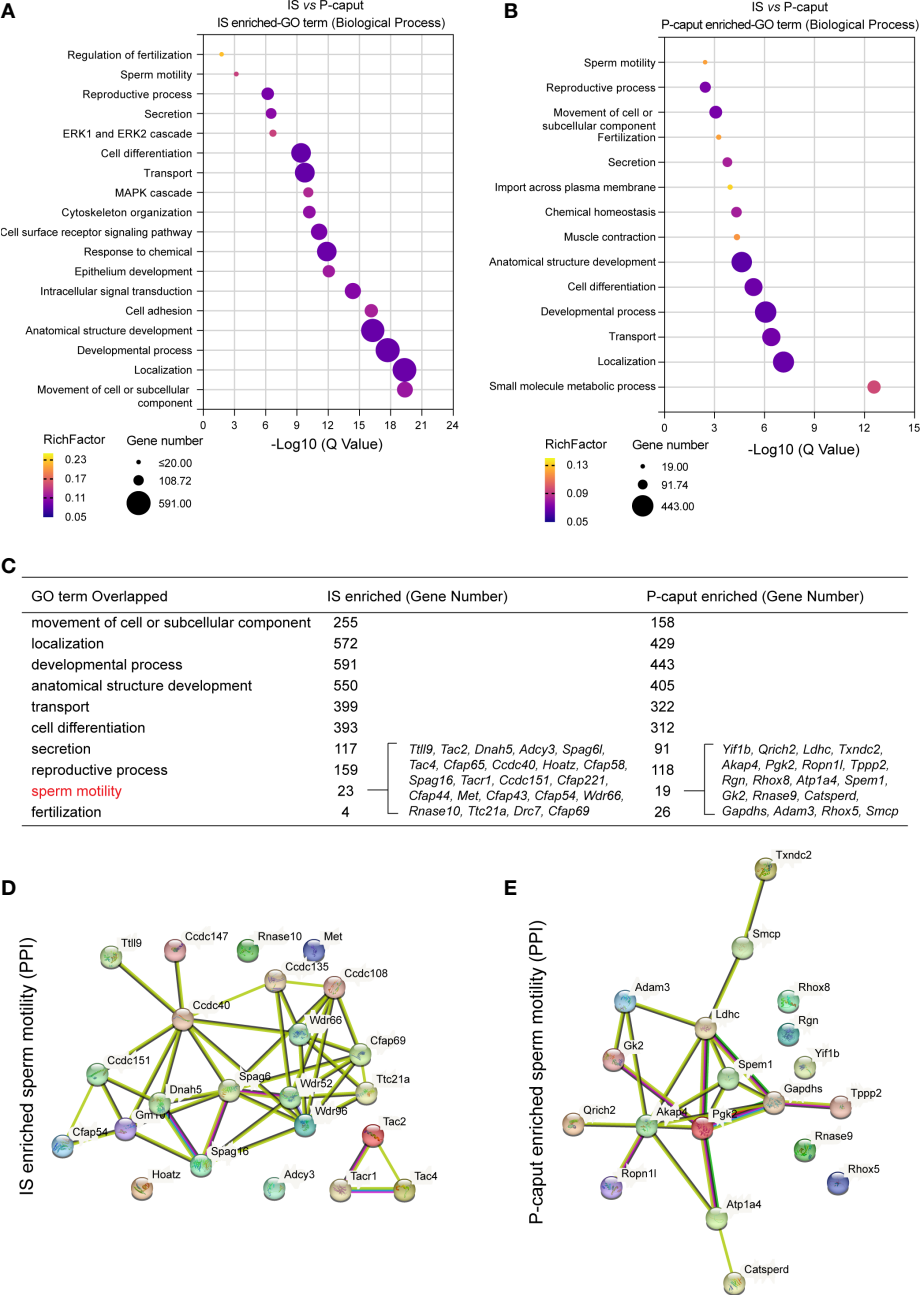
Gene Ontology enrichment analysis of region-specific genes. **(A, B)** Gene Ontology (GO) terms of biological process for IS enriched transcripts **(A)** and P-caput enriched transcripts **(B)**. **(C)** Comparisons of the enriched GO terms for the IS abundant and P-caput abundant genes. **(D, E)** Twenty-three IS enriched genes **(D)** and 19 P-caput enriched genes **(E)** involved in sperm motility were plotted in the protein–protein interaction (PPI) networks based on confidence scores using STRING database.

GO was used to identify biological processes of DEGs (**Figures 3A, B**). We compared the enriched GO terms for the IS and P-caput specific genes and found many similar biological processes. Notably, the overlapping GO terms are associated with cell or subcellular component movements, their localization, developmental processes, transport, cell differentiation, secretion, reproductive processes, sperm motility, and fertilization (**Figure 3C**). Moreover, the 23 IS enriched genes and 19 P-caput enriched genes involved in sperm motility were selected and plotted in the PPI networks based on a high confidence score from the STRING database. The PPI networks documented potential interactions among these sperm motility-related proteins in the IS and P-caput (**Figures 3D, E**).

Collectively, our RNA-seq analysis identified a series of region-specific genes and some of the top DEGs could be used as potential markers for the IS and P-caput. In addition, our dataset documented that region-specific genes were associated with transport, secretion, sperm motility, fertilization, and male fertility. This suggested that the segment-specific epididymal microenvironments were important mediators of these biological processes and these region-specific genes had similar functions during sperm maturation and male fertility.

### 3.3 Verification of region-specific genes

**Figure 4:**
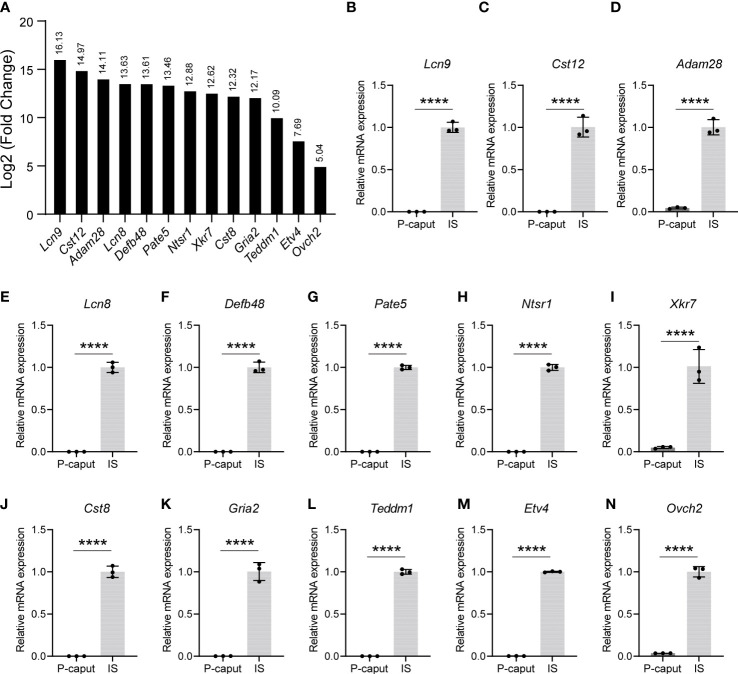
Selection and verification of the IS abundant genes. **(A)** RNA-seq results of several selected transcripts (log2 fold change) that were at the top of the IS enriched gene list. **(B–N)** Quantitative RT-PCR analysis of selected genes. The data showed that these genes were specifically expressed in the IS. The IS expressed genes relative to *β-actin* were set to 1. Data are presented as mean ± SD for *n* = 3 biologically independent experiments. ****p < 0.0001 by two-tailed Student’s *t*-test.

**Figure 5:**
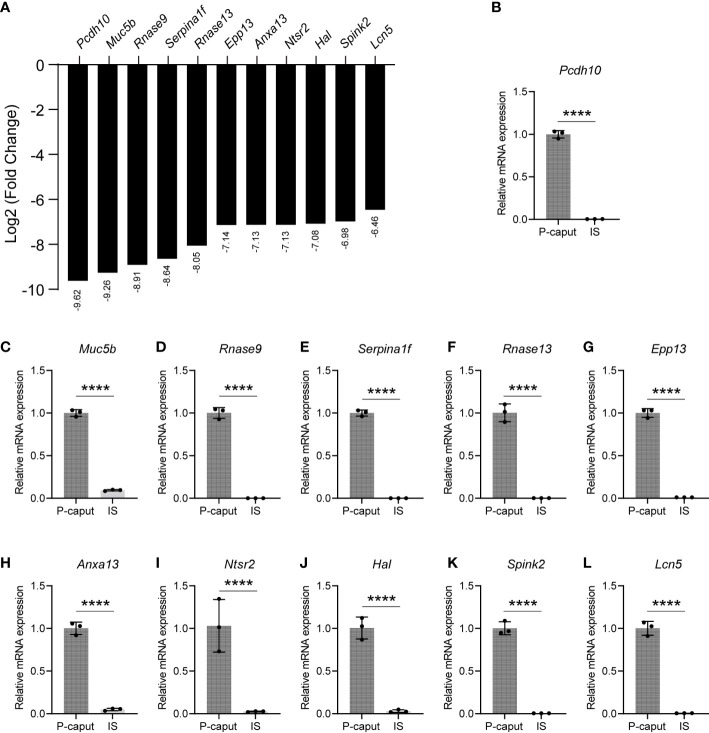
Selection and validation of the P-caput abundant genes. **(A)** A set of 11 transcripts (log2 fold change) that were at the top of the P-caput enriched gene list were selected based on RNA-seq data. **(B–L)** Quantitative RT-PCR experiments were performed to validate the expression patterns of these selected genes. The data showed that these genes were abundantly expressed in the P-caput. The P-caput expressed genes relative to *β-actin* were set to 1. Data are presented as mean ± SD for *n* = 3 biologically independent experiments. ****p < 0.0001 by two-tailed Student’s *t*-test.

Based on our RNA-seq analysis, 1,961 transcripts were abundantly expressed in the IS. As shown in **Figure 4A**, we selected some of the most abundant or previously defined IS genes from the RNA-seq data including *Lcn9*, *Cst12*, *Adam28*, *Lcn8*, *Defb48*, *Pate5*, *Ntsr1*, *Xkt7*, *Cst8*, *Gria2*, *Teddm1*, *Etv4*, and *Ovch2*. We verified the expression patterns of these transcripts by real-time RT-PCR, which confirmed specific expression in the IS (**Figures 4B–N**). The RNA-seq analysis also identified 1,739 transcripts that were expressed in the P-caput and included *Pcdh10*, *Muc5b*, *Rnase9*, *Serpina1f*, *Rnase13*, *Epp13*, *Anxa13*, *Ntsr2*, *Hal*, *Spink2*, and *Lcn5* (**Figure 5A**). P-caput expression of all was authenticated by qRT-PCR (**Figures 5B–L**). Overall, we selected and validated a series of region-specific genes in the IS and P-caput and propose that these genes can be used as regional markers in the epididymis.

### 3.4 Validation of DEGs in a region-dependent manner

Many genes expressed in the epididymis have been well-characterized. *Adam28* and *Cst8* have been shown to be mainly expressed in the IS by microarray and qRT-PCR (12). *In situ* hybridization shows abundant *Teddm1* mRNA expression in the IS and abundant *Serpina1f* mRNA expression in the P-caput of mice (25) (**Supplementary Figures S3A, E**). In addition, a recent RNA-seq study reports that *Adam28*, *Defb48*, and *Teddm1a* are expressed in the mouse IS, whereas *Epp13* and *Rnase13* are primarily expressed in the caput (22). Furthermore, LCN8, CST12, and OVCH2 proteins are localized in the IS by reporter mouse lines and immunostaining, and RNASE9 has a strong signal in rat P-caput by immunohistochemistry (26–29) (**Supplementary Figures S3B–D, F**). Thus, the region-specific expression of these genes in our RNA-seq and qRT-PCR data agrees with the protein or transcriptional patterns reported in the literature. These comparisons validate not only the accuracy of our microdissection but also the RNA-seq results.

**Figure 6:**
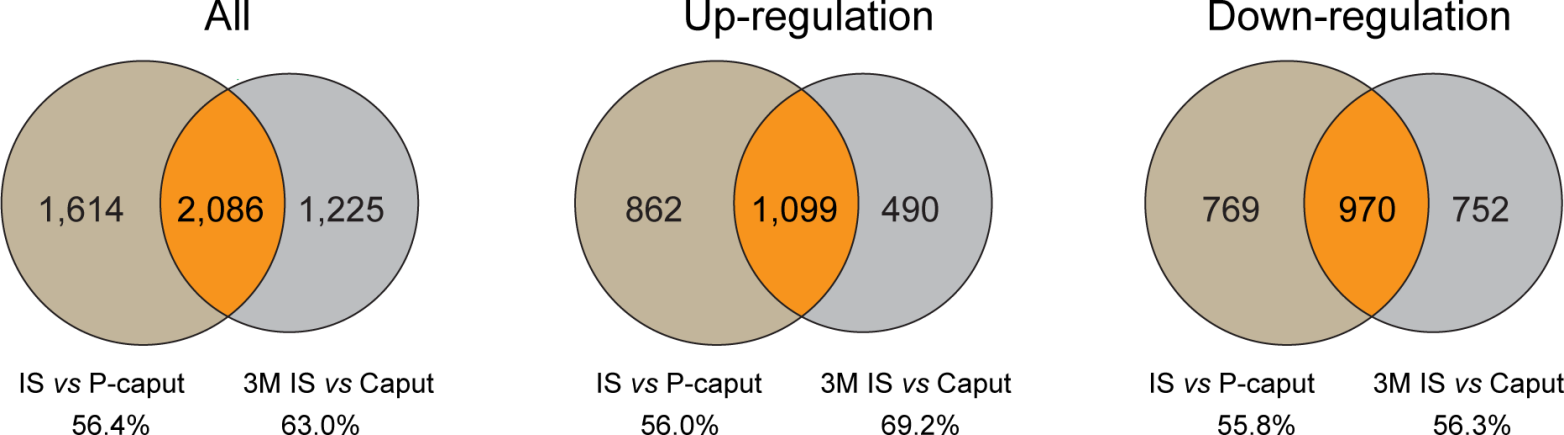
Comparison of published RNA-seq data of IS and caput with our RNA-seq data. Published RNA-seq data of 3 months IS and caput were analyzed using the same criteria of adjusted *p*-value <0.05 and log2 (fold change) >1 by DESeq2. Venn diagram showing the overlapping DEGs of all, upregulated, and downregulated transcripts in IS. The number and percentage of overlapping DEGs were shown.

To further highlight the integrity and reliability of our RNA-seq data, published RNA-seq data of 3-month IS and caput were analyzed using the same criteria of adjusted *p*-value <0.05 and log2 (fold change) >1 by DESeq2 and compared with our RNA-seq data. Venn diagram displayed the overlapping DEGs of all, upregulated, and downregulated transcripts in the IS (**Figure 6**). The total of 2,086 DEGs that were shared represent 56.4% (3,700) and 63.0% (3,311), respectively, of our data and published RNA-seq data. The 1,099 upregulated genes that were shared represent 56.0% (1,961) and 69.2% (1,589), respectively, of our data and published data. The 970 transcripts that were decreased in both data reflect 55.8% (1,739) and 56.3% (1,722) of downregulated genes in our data and published RNA-seq data, respectively. These data suggest that a significant overlap of DEGs was observed in our data and published RNA-seq data, reflecting the reliability of our RNA-seq analysis.

### 3.5 Region-specific genes are associated with sperm maturation and male fertility

The effect on sperm maturation and male fertility of many epididymal genes has been characterized. Here, we selected several DEGs from our RNA-seq data and categorized them into either epididymis-enriched or epididymis-specific based on published literature. As shown in **Table 1**, the IS abundant genes, *Lcn9*, *Lcn8*, *Pate5*, and *Ovch2*, were determined to be epididymis-specific (23, 26, 28, 30, 32), and *Cst8*, *Cst12*, *Adam28*, and *Etv4* were epididymis-enriched (28, 32, 33). As shown in **Table 2**, P-caput abundant genes, *Rnase9*, *Rnase13*, *Epp13*, *Serpina16*, and *Serpina1f*, were epididymis-specific (25, 35–37), and *Pcdh10*, *Muc5b*, *Ntsr2*, *Spink2*, *H3c15*, *Defb39*, *Hal*, *Slc6a18*, and *Lcn5* were epididymis-enriched (31, 38, 39). Among these IS abundant genes, *Lcn8*, *Lcn9*, and *Adam28* knock out (KO) mice had normal spermatogenesis and male fertility, whereas *Lcn8* KO males displayed abnormal sperm morphology, decreased motility, and increased spontaneous acrosome exocytosis. *Etv4* KO mice exhibited normal spermatogenesis and sperm morphology but abnormal ejaculation (**Table 1**) (28, 30, 31, 33). Male mice lacking *Cst* family genes (deleted region between *Cstl1* and *Cstdc2*, which includes *Cst8* and *Cst12*) had normal sperm morphology and motility, but spermatozoa bound poorly to the zona pellucida (ZP) and could not pass through the utero-tubal junction (UTJ), resulting in subfertility (**Table 1**) (32). Male mice lacking *Pate* family genes (deleted region between *Pate8* and *Pate10*, which includes *Pate5*) displayed a similar phenotype (**Table 1**) (32). *Ovch2* KO males were completely sterile because sperm had defects in ADAM3 processing, the ability to bind to the zona pellucida and passage through the UTJ (**Table 1**) (28). In summary, mutations of *Cst* family genes, *Pate* family genes, and *Ovch2* expressed in the IS region cause severe male fertility defects due to lack of mature membrane protein ADAM3 on the sperm heads, which is thought to play a pivotal role in sperm–zona pellucida binding and sperm migration through the UTJ. These data suggest that genes mutated in the IS region have a high potential for causing severe defects in sperm motility, ADAM3 processing, and male fertility.

**Table 1:** Summary of several top IS abundant genes in male reproduction.

Gene	Epididymis-specific	IS-abundant	KO model	Reproductive phenotype	References
*Lcn9*	Yes	Yes	*Lcn9* KO	Normal spermatogenesis and fertility; normal sperm	(30, 31)
*Lcn8*	Yes	Yes	*Lcn8* KO	Normal spermatogenesis and fertility; abnormal sperm; decreased sperm motility; increased acrosome reaction frequencies	(26, 30)
*Cst12*	No	Yes	*Cstl1-Cstdc2* KO	Subfertility; normal sperm morphology and motility; spermatozoa barely bound the zona pellucida; spermatozoa could not pass through the utero-tubal junction	(27, 32)
*Cst8*	No	Yes	*Cstl1-Cstdc2* KO	Subfertility; normal sperm morphology and motility; spermatozoa barely bound the zona pellucida; spermatozoa could not pass through the utero-tubal junction	(12, 32)
*Pate5*	Yes	Yes	*Pate8-Pate10* KO	Severe subfertility; normal sperm morphology and motility; spermatozoa barely bound the zona pellucida and could not pass through the utero-tubal junction	(32)
*Ovch2*	Yes	Yes	*Ovch2* KO	Sterility; sperm could not pass through the utero-tubal junction and bind to the zona pellucida; OVCH2 is required for sperm ADAM3 processing	(28)
*Etv4*	No	Yes	*Etv4* KO	Normal spermatogenesis and sperm morphology; abnormal ejaculation	(33, 34)
*Adam28*	No	Yes	*Adam28* KO	Normal fertility	(12, 28)

**Table 2:** Summary of several top P-caput abundant genes in male reproduction.

Gene	Epididymis-specific	P-caput-abundant	KO model	Reproductive phenotype	References
*Pcdh10*	No	Yes	*Pcdh10* KO	NA	NA
*Muc5b*	No	Yes	*Muc5b* KO	NA	NA
*Ntsr2*	No	Yes	*Ntsr2* KO	NA	NA
*Serpina1f*	Yes	Yes	NA	NA	(25)
*H3c15*	No	Yes	NA	NA	NA
*Defb39*	No	Yes	NA	NA	NA
*Hal*	No	Yes	*Hal* mutant	NA	NA
*Slc6a18*	No	Yes	*Slc6a18* KO	NA	NA
*Serpina16*	Yes	Yes	*Serpina16* KO	Normal fertility; normal sperm morphology and motility	(35)
*Rnase9*	Yes	Yes	*Rnase9* KO	Normal spermatogenesis and fertility; normal sperm morphology; impaired sperm maturation	(29, 36)
*Rnase13*	Yes	Yes	*Rnase13* KO	Normal fertility; normal sperm morphology	(37)
*Epp13*	Yes	Yes	*Epp13* KO	Normal fertility; normal sperm morphology	(37)
*Spink2*	No	Yes	*Spink2* KO	Azoospermia; abnormal sperm morphology; decreased sperm motility	(38, 39)
*Lcn5*	No	Yes	Quadruple/quintuple KO	Subfertility; normal sperm morphology; sperm barely bound to the zona pellucida	(31, 40, 41)

NA, not applicable.

No KO mice or no reproductive phenotypes were reported in literatures currently.

Among the P-caput abundant genes, reproductive phenotypes have not been reported for *Pcdh10*, *Muc5b*, *Slc6a18*, *Ntsr2* KO, and *Hal* mutant males, and no KO mouse model has been reported for *H3c15*, *Defb39*, and *Serpina1f* (**Table 2**). *Rnase9*, *Rnase13*, *Serpina16*, and *Epp13* KO mice demonstrated normal fertility and normal sperm morphology, but *Rnase9* KO males showed impaired sperm maturation (**Table 2**) (35–37). *Spink2* KO mice had azoospermia, abnormal sperm morphology, and decreased sperm motility (**Table 2**) (39). Quadruple KO (*Lcn5*, *Lcn6*, *Lcn8*, and *Lcn10*) or quintuple KO (*Lcn5*, *Lcn6*, *Lcn8*, *Lcn10*, and *Lcn9*) male mice demonstrated normal sperm morphology, but the sperm barely bound to the zona pellucida because of defective ADAM3 processing (**Table 2**) (31). These findings suggest that mutations of the lipocalin family genes (e.g., *Lcn5*) or other genes (e.g., *Spink2*) expressed in P-caput are possibly related with aberrant ADAM3 processing, or sperm motility defects and male fertility defects.

Taken together, these data, together with the GO term analysis results, suggest that genes mutated in the IS or P-caput are potentially associated with similar sperm maturation defects including motility and fertilizing ability. Thus, the segment-specific epididymal microenvironments must play an important role in sperm maturation, motility, and fertility.

## 4 Discussion

Previous studies have shown that epididymal aging occurs in a region-dependent manner and the proximal region is particularly vulnerable because of its rich blood supply (22, 42). It also has been suggested that both cauda and caput epididymal sperm can support full-term development in FVB and CD-1 mice (43). These findings indicate that the proximal region of the epididymis plays an important role during sperm transition and maturation. Therefore, the IS and caput epididymis were the focus of our detailed investigations.

In this study, the segment-dependent gene expression patterns of the mouse caput epididymis were profiled by RNA-seq. The segments used for the transcriptome analysis in the current study had to be accurately identified and isolated. Histology-based epididymal segment determination has been used before (12, 44), but the *Lcn9*-*cre*; *Rosa26^tdTomato^* mice facilitated precise microdissection to physically isolate the IS and P-caput. Specifically, the IS was labeled by red fluorescence and the P-caput region was defined by connective tissue septae. This experimental approach allowed us to obtain accurate segments and their specific transcriptomes. A series of studies have focused on the transcriptome changes in the epididymis. However, they have been limited either in technology (microarray) or in conventional segmentation approaches. In the current study, the caput epididymis was accurately subdivided into the IS and P-caput by using *Lcn9*-*cre*; *Rosa26^tdTomato^* mice. Our RNA-seq analysis established distinct transcriptional profiles for the IS and P-caput and identified a series of region-specific genes that were potentially involved in sperm maturation and male fertility. GO term analysis was conducted on the IS and P-caput enriched transcripts and revealed that 23 genes and 4 genes were involved in sperm motility and fertilization, respectively, in IS, and 19 genes and 26 genes were enriched in sperm motility and fertilization, respectively, in P-caput. In addition, a total number of 159 IS enriched genes and 118 P-caput enriched genes were associated with reproductive process. For example, the lipocalin family quintuple KO (*Lcn5* and *Lcn9* included) males were mostly infertile with a reduced amount of mature ADAM3, which is an essential protein for sperm binding to the zona pellucida. The quintuple KO spermatozoa lack sperm plasma membrane proteins CMTM2A/B, suggesting that lipocalins affect ADAM3 localization and precursor processing through CMTM2A/B on the sperm heads. OVCH2, as a secreted protease, is essential for sperm surface ADAM3 processing. *Ovch2* KO males are sterile due to aberrant ADAM3 processing on the sperm heads, which, in turn, causes the failure of sperm binding to the zona pellucida. Altogether, these data suggest that IS- or P-caput-expressing genes play a critical role in sperm maturation and male fertility.

In our RNA-seq analysis, we found that 1,961 genes were abundantly expressed in the IS and 1,739 genes were prominently expressed in the P-caput. The top IS genes included *Lcn9*, *Cst12*, *Adam28*, *Lcn8*, *Defb48*, *Pate5*, *Ntsr1*, *Xkt7*, *Cst8*, *Gria2*, *Pate10*, *Teddm1*, *Etv4*, and *Ovch2*. The top P-caput genes included *Pcdh10*, *Muc5b*, *Rnase9*, *Serpina1f*, *Rnase13*, *Epp13*, *Anxa13*, *Ntsr2*, *Hal*, *Spink2*, and *Lcn5*. These most abundant DEGs could be potential markers of the IS or P-caput. Among these region-specific genes, many of them are predominantly or uniquely expressed in the epididymis including *Lcn9*, *Lcn8*, *Pate5*, *Pate10*, *Ovch2*, *Rnase9*, *Rnase13*, *Serpina16*, *Serpina1f*, and *Epp13*. Also, many of the region-specific genes are associated with transport, secretion, sperm motility, and male fertility.

A number of top DEGs defined by RNA-seq data in this study were validated by qRT-PCR. The relative mRNA levels determined by both qRT-PCR and RNA-seq analysis were very similar for all 24 genes examined. Moreover, our RNA-seq data were consistent with previous published data of either gene or protein expression in mice and rats for *Lcn9* (23), *Lcn8* (26), *Adam28* (12, 22), *Defb48* (22), *Teddm1* (22, 25), *Cst12* (27), *Cst8* (12), *Etv4* (34), *Ovch2* (28), *Rnase9* (29), *Rnase13* (22), *Epp13* (22), *Serpina1f* (25), and *Lcn5* (40, 41). For example, *Adam28* mRNA has been shown to be abundantly expressed in the proximal region of the mouse epididymis (corresponding to the IS) by qRT-PCR and RNA-seq analyses (12, 22). Additionally, LCN8, CST12, and OVCH2 proteins are in the IS region in reporter mouse line and detected by immunostaining (26–28). *Serpina1f* mRNA demonstrates a strong signal in the mouse P-caput by *in situ* hybridization analysis and RNASE9 protein displays a strong signal in the rat P-caput by immunohistochemistry (25, 29). The results of our qRT-PCR and RNA-seq analyses demonstrated that *Adam28*, *Lcn8*, *Cst12*, and *Ovch2* were predominantly expressed in the IS, while *Serpina1f* and *Rnase9* were primarily expressed in the P-caput. However, many of the DEGs identified in our RNA-seq data are still needed to verify their regional specificity at protein levels by immunostaining assay in the future. Collectively, our findings, together with the results available in the literature, demonstrate the precision of our microdissection and the accuracy of our RNA-seq analyses.

Recently, several scRNA-seq studies provided an atlas of cell composition across the epididymis, and defined spatiotemporal differences in DEGs in mouse and human epididymal segments (21, 45, 46). These investigations documented transcriptional signatures for multiple cell clusters and identified individual roles for principal cells, apical cells, narrow cells, basal cells, clear cells, halo cells, and stromal cells in the epididymis. Furthermore, the transcriptome atlas of adult and aged mouse epididymides has been characterized recently (22). The study characterized age-related transcriptome changes in all seven regions (testis, efferent ductules, IS, caput, corpus, cauda, and ductus deferens) of the male reproductive tract by comparing RNA-seq datasets from the 3-month and 21-month mice. There were 475 upregulated and 902 downregulated age-related DEGs in IS, and 687 upregulated and 865 downregulated transcripts in aged caput, suggesting age-related transcriptional changes in proximal epididymis. The upregulated DEGs in IS and caput were largely associated with T-cell activation, indicating increased immune response activities in aged mice. Downregulated DEGs in IS were mainly enriched for spermatid development, and those in caput were primarily related to extracellular matrix organization, reflecting degenerative changes of the structures in aged IS and caput. Altogether, our data, together with previous studies, provided a roadmap and a wealth of molecular hypotheses for future efforts in sperm maturation and reproductive physiology.

In conclusion, our investigations establish a robust gene expression dataset and provide an RNA-seq resource for identification of region-specific genes in the caput epididymis. These epididymal selective/specific genes may prove to be targets for male contraception or may provide new insights into understanding sperm transport, maturation, and male fertility. To gain in-depth understanding of epididymal function and epididymis diseases, functional experiments of the genes identified in this study are needed in the future.

## Data availability statement

The data presented in the study are deposited in the NCBI Sequence Read Archive (SRA) repository, accession number PRJNA915466.

## Ethics statement

The animal study was reviewed and approved by Ethics Committee for Animal Research of School of Life Sciences, Shandong University.

## Author contributions

ZW conceived and designed the study and analyzed most of the data. XW, FQ, JY, and MZ performed the experiments. AZ and XX analyzed the published RNA-seq data. X-YS provided the mice for the study. ZW wrote and edited the manuscript. All authors contributed to the article and approved the submitted version.
