# Supplementary material for: Transcriptome profiling of the initial segment and proximal caput of mouse epididymis
Source: Front Endocrinol (Lausanne). 2023 May 31;14:1190890. doi: 10.3389/fendo.2023.1190890 (PMC10266198; doi:10.3389/fendo.2023.1190890)
Supplement: Supplementary file 1 [file Presentation_1.pdf]

Supplementary Material

Transcriptome profiling of the initial segment and proximal caput of mouse epididymis

Xiao Wang[1†], Fanyi Qiu[1†], Junjie Yu[1], Meiyang Zhou[1], Anjian Zuo[2], Xiaojiang Xu[3], Xiao-Yang Sun[1] and Zhengpin Wang[1]*

*** Correspondence:** Zhengpin Wang: zhengpin.wang@sdu.edu.cn

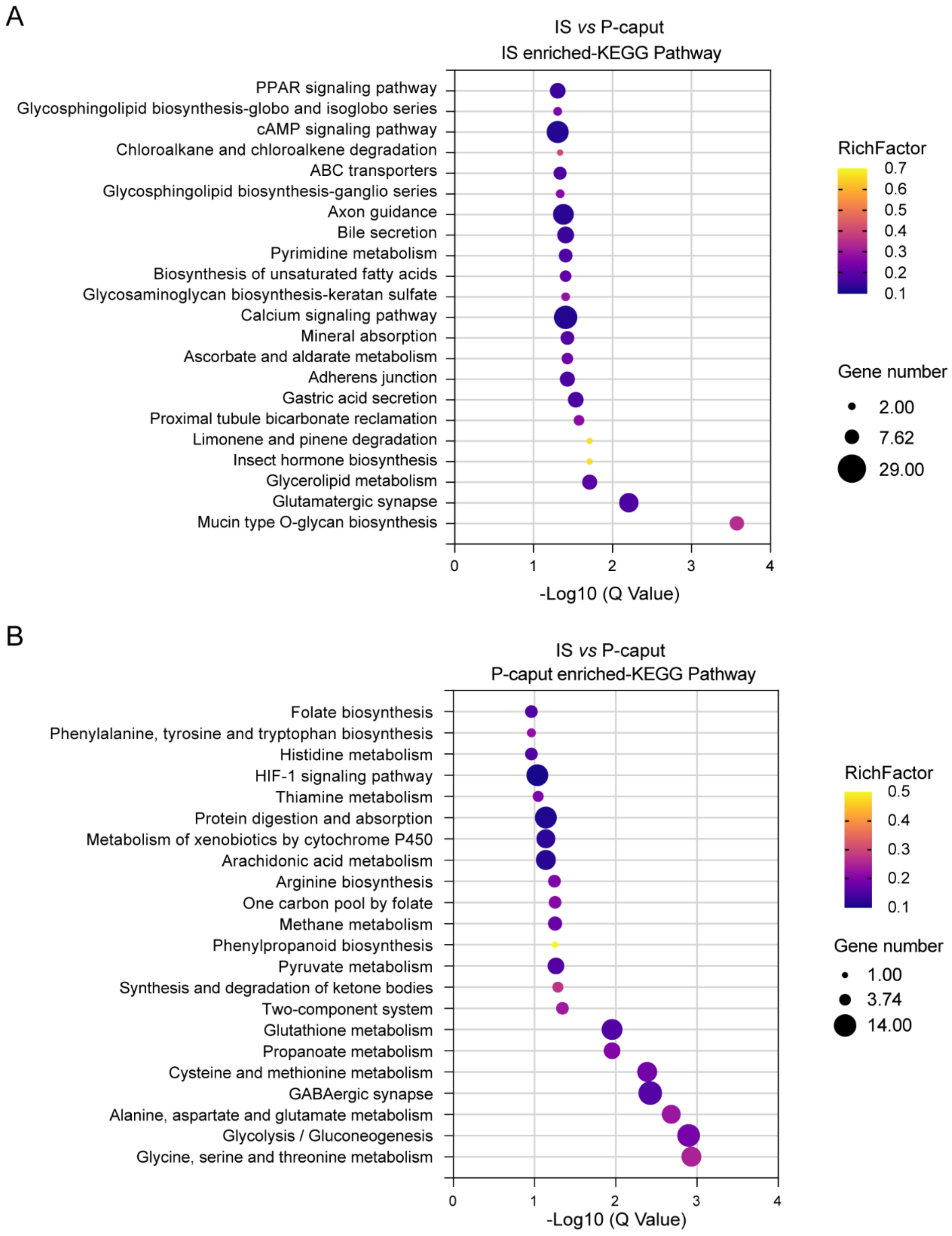

Supplementary Figure 1. Kyoto Encyclopedia of Genes and Genomes (KEGG) enrichment analysis of region-specific genes. (A) KEGG terms for IS enriched transcripts. (B) KEGG analysis showing the enriched pathways for P-caput enriched transcripts.

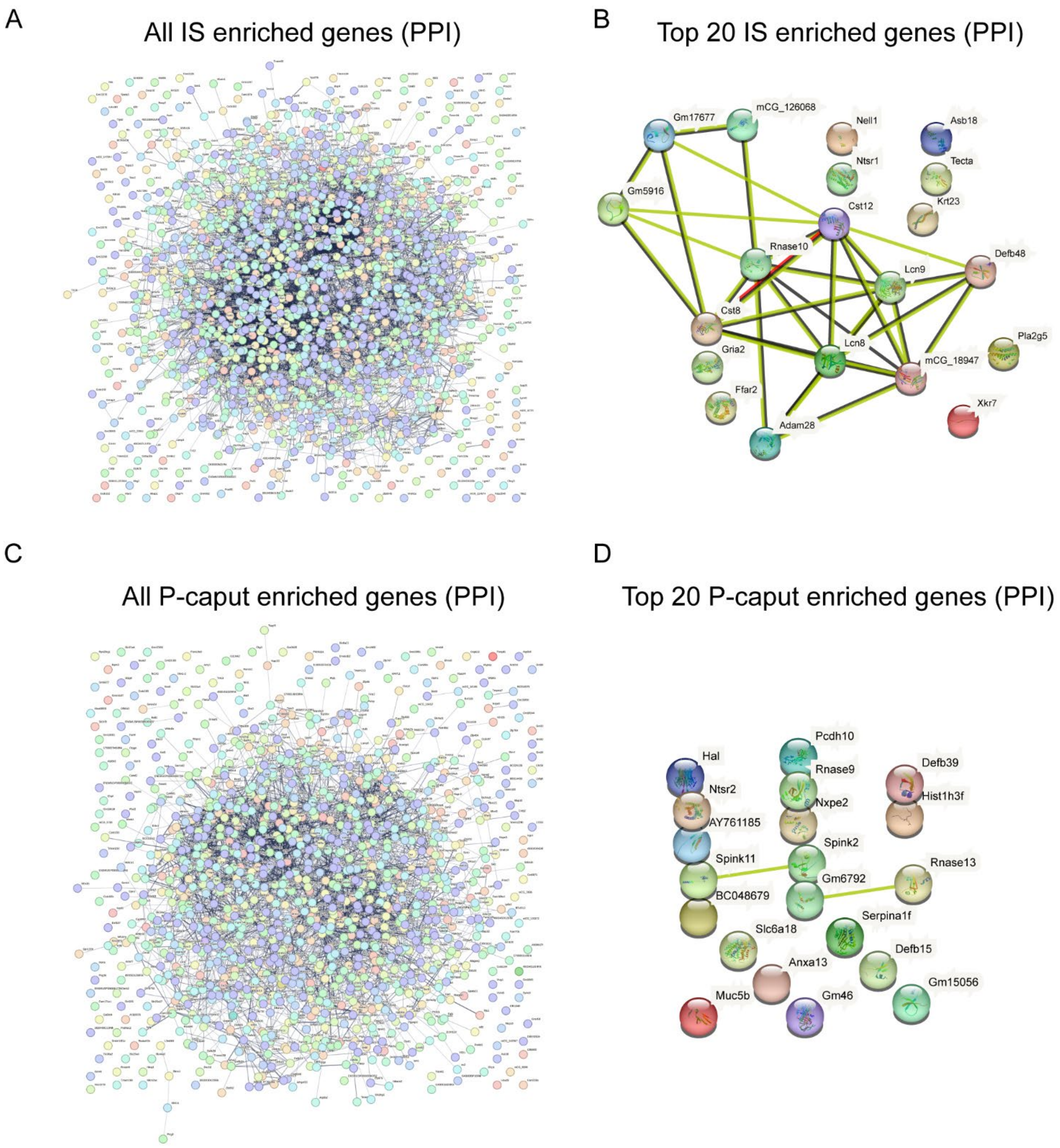

Supplementary Figure 2. Protein-protein interaction (PPI) networks of DEGs. (A) The PPI networks of all IS enriched DEGs. (B) The PPI networks of the top 20 IS enriched transcripts. (C) The PPI networks of all P-caput enriched DEGs. (D) The PPI networks of the top 20 P-caput enriched genes.

IS-enriched

A

Teddm1

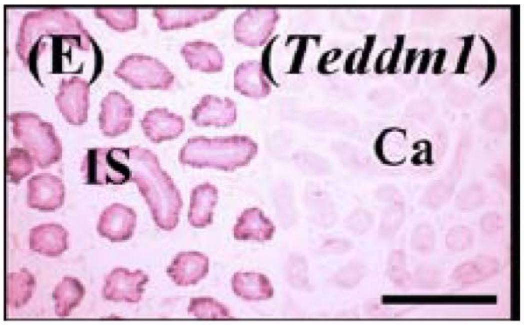

In situ hybridization analysis shows abundant *Teddm1* mRNA expression in the mouse IS.
Adapted from Yamazaki, et al; 2006

B

LCN8

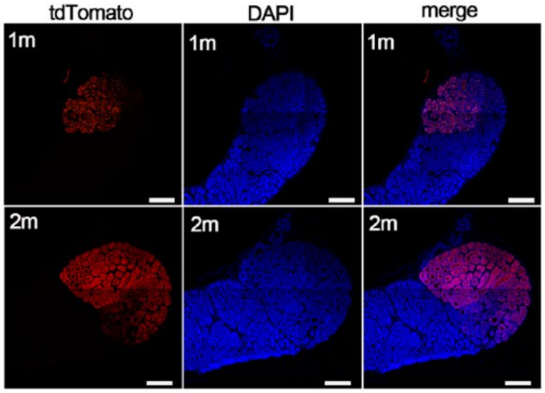

Lcn8-cre; tdTomato Epididymis
LCN8 is expressed in the mouse IS.
Adapted from Gong, et al; 2021

C

CST12

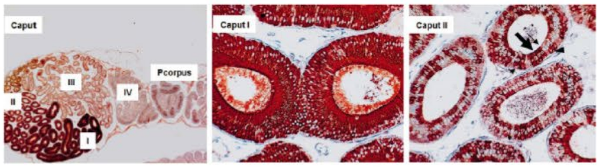

CST12 is strongly expressed in the mouse IS.
Adapted from Li, et al; 2005

IS-enriched

D

OVCH2

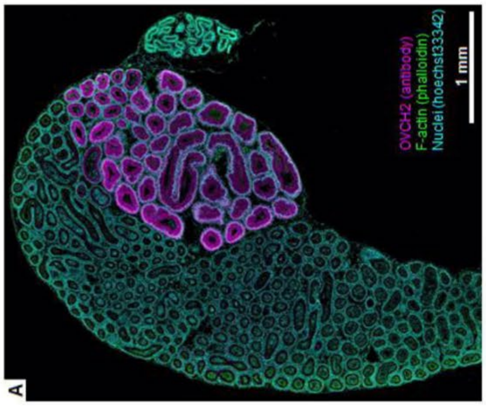

OVCH2 is expressed in the mouse IS.
Adapted from Kiyozumi, et al; 2020

E

P-caput-enriched

Serpina1f

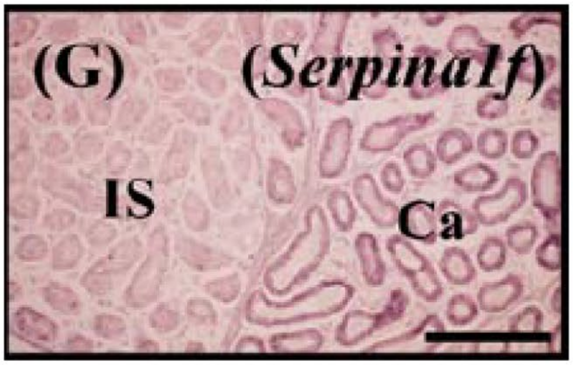

In situ hybridization analysis shows abundant *Serpina1f* mRNA expression in the mouse proximal caput.
Adapted from Yamazaki, et al; 2006

F

RNASE9

IS P-caput

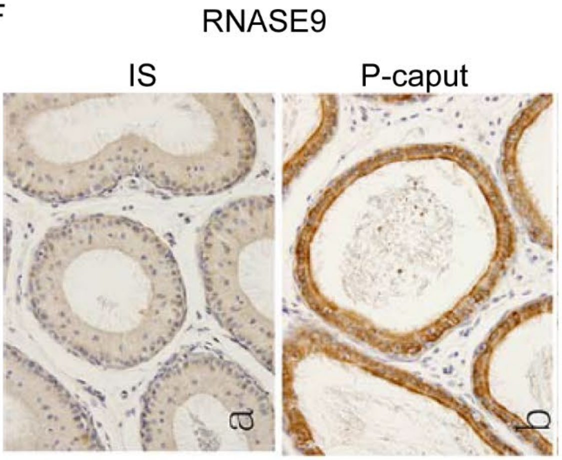

RNASE9 is stronly expressed in the rat proximal caput.
Adapted from Zhu, et al; 2007

Supplementary Figure 3. *In situ* hybridization and immunostaining data adapted from the published literature. (A) *In situ* hybridization of *Teddm1* in the mouse epididymis adapted from Yamazaki, et al; 2006. (B) tdTomato signals in the mouse epididymis from *Lcn8-cre*; $Rosa26^{tdTomato}$ mice adapted from Gong, et al; 2021. (C) Immunohistochemistry of CST12 protein in the mouse epididymis adapted from Li, et al; 2005. (D) Immunofluorescence of OVCH2 in the mouse epididymis adapted from Kiyozumi, et al; 2020. (E) *In situ* hybridization of *Serpina1f* in the mouse epididymis adapted from Yamazaki, et al; 2006. (F) Immunohistochemistry of RNASE9 protein in the rat epididymis adapted from Zhu, et al; 2007.

Supplementary Table 1. Primers used in this study.

Gene	Direction	Primers (5'-3')
Lcn9-cre (WT) (Genotype)	F1[A]	CTGCAATCTATATGGGCTGGACTC
	R2	CAGGGTAGTTTCCCACTCTTCTCAG
Lcn9-cre (Genotype)	F1	CTGCAATCTATATGGGCTGGACTC
	R1	TTTTCTTCAACATCTCCTGCTTGC
tdTomato (WT) (Genotype)	F	AAGGGAGCTGCAGTGGAGTA
	R	CCGAAAATCTGTGGGAAGTC
tdTomato (Mutant) (Genotype)	F	GGCATTAAAGCAGCGTATCC
	R	CTGTTCCTGTACGGCATGG
Adam28	F	AAGAGCCAAAGTTCCTTTTGTCA
	R	CACGCGGCCTATTTGCTGA
Lcn8	F	GCTAGGCTGCTGAGCAATGT
	R	CCAGTTTTTGGTCAGAAGCAAC
Defb48	F	ATAGAAGACGAGATCGGTGCT
	R	GTCTGAGAAGATAACTGGCGTTG
Pate5	F	TGCTCCTACACTTTCTGTTGATG
	R	CTTGCCTACCAAACAATGTCCA
Ntsr1	F	CAGTTCGGACTGGAGACGATG
	R	ACCAGCACCTTGGAATAAATGTC
Xkr7	F	GCGCTGCTCGTGTTCTTCT
	R	TGAGGCCGAAGTAGGTGCT
Cst8	F	GTGTTTGGTTTGCCATGAAAGAA
	R	TGGTATTCCATTCGGTCTGTGAT
Gria2	F	GCCGAGGCGAAACGAATGA
	R	CACTCTCGATGCCATATACGTTG
Teddm1	F	CCCGGCTGTGGAAAATAGC
	R	GTGGCACCCCTTTGGTCAT
Etv4	F	CGCACAGACTTCGCCTACG
	R	CAGACATCATCTGGGAATGGTC
Ovch2	F	TCTGCTTGGAACAAGGTCATTC
	R	GAGGTTGGGGCTTAACCAAAC
Pcdh10	F	AGCTCTAAGGACAGTGGTCAT
	R	CAGTGCTTTACATTCCTCGGT
Muc5b	F	GTGGCCTTGCTCATGGTGT
	R	CGCTCATGCTAGGGAAGACAG
Rnase9	F	TATAAGGGGCGCTGTTACCCA
	R	TCTGCAAGATTTAACGCCATTCT
Serpina1f	F	TGGCTCTTACCATCTGCAATG
	R	GCAATGACTCTAATCGGGGAGA
Rnase13	F	TTGTCCTCCCGCTTGTCTTC
	R	CCATTACAGTATCCGTGGAATCC
Epp13	F	GACCAGTGGATGGAACTTTCTG
	R	GGCAGTAACACCAGTCTCCTTT
Anxa13	F	GGCCAGTCGTGATGAAGAAGA

	R	TTGAAGGCAAGTTCGTCAGTG
Ntsr2	F	TTCACCGCGCTCTATTCGC
	R	AGGGGTAGTGGGACCACAC
Hal	F	CTGTGCGACGCTACATGAAGA
	R	TCATTGTCCTCTAAGGCCACC
Spink2	F	CATGAGACTCTCGACTCTTCCG
	R	CGCACACAGGGTTGAGGTT
Lcn5	F	CTCCAAGATGGGTGCATACGG
	R	CCTCATTGTAATAGGTGGTGGTC
β-actin	F	GGCTGTATTCCCCTCCATCG
	R	CCAGTTGGTAACAATGCCATGT

[A]F, forward; R, reverse
